# Genetic screens using the *piggyBac* transposon

**DOI:** 10.1016/j.ymeth.2010.12.022

**Published:** 2011-04

**Authors:** Su Kit Chew, Roland Rad, P. Andrew Futreal, Allan Bradley, Pentao Liu

**Affiliations:** Wellcome Trust Sanger Institute, Wellcome Trust Genome Campus, Hinxton, Cambridge CB10 1HH, UK

**Keywords:** Transposon, Transposition, *piggyBac*, Mouse, Mutagenesis, Cancer

## Abstract

Transposons are an attractive system to use in genetic screens as they are molecularly tractable and the disrupted loci that give rise to the desired phenotype are easily mapped. We consider herein the characteristics of the *piggyBac* transposon system in complementing existing mammalian screen strategies, including the *Sleeping Beauty* transposon system. We also describe the design of the *piggyBac* resources that we have developed for both forward and reverse genetic screens, and the protocols we use in these experiments.

## 1 Introduction

Genetic screens in model organisms represent a stalwart means of gene function discovery. Class II transposable elements or DNA transposons are mobile elements that can relocate between genomic loci via a ‘cut and paste’ mechanism, they are proven workhorses in the molecular toolkit of diverse genetic models from the fruitfly to maize [1,2]. DNA transposons comprise two principle components, the transposase enzyme that mediates the transposition process itself and the inverted terminal repeats flanking the transposition cargo that the transposase recognises. This relocation activity of DNA transposons has been exploited to great effect in *Drosophila* for diverse purposes, ranging from transgene delivery to mutagenesis to chromosome engineering [3].

Two transposition systems developed for use in mammalian systems have come to the fore in recent years – *Sleeping Beauty* (*SB*) and *piggyBac* (*PB*). *Sleeping Beauty* (*SB*) was engineered from Tc1/mariner transposon fossils in salmonid fish genomes by comparative phylogenetic analysis [4]. More recently, *piggyBac* (*PB*), which was originally characterised in insects, was found to transpose efficiently in the mammalian cells [5–7].

### 1.1 *piggyBac* traits and considerations in experiments

**Fig. 1:**
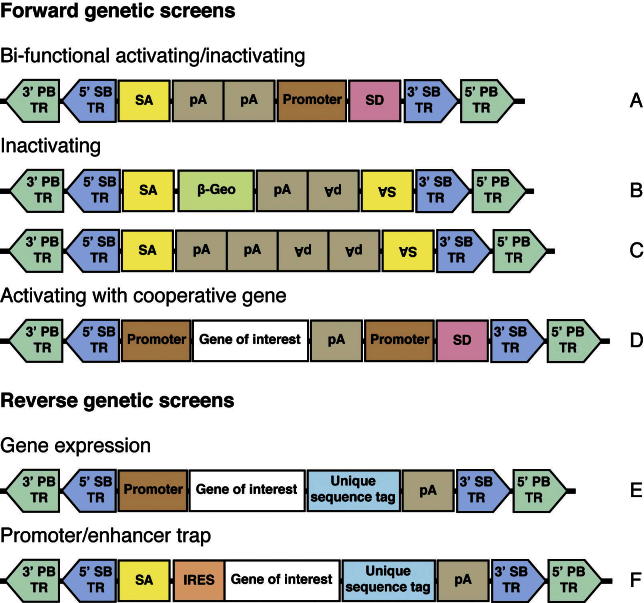
Catalogue of transposon designs useful for different genetic screens. They all have PB inverse terminal repeats to facilitate delivery and nested SB within it for flexibility in subsequent mobilisation (TR, terminal repeat; SA, splice acceptor; pA, polyadenylation sequence; SD, splice donor; IRES, internal ribosomal entry site).

*PB* transposase recognises a short TTAA motif in the host genome for insertion and excises the transposon without mutation [6,8]. In mammalian cells, due to the different transposition characteristics of *Sleeping Beauty* and *piggyBac*, it can be advantageous to use both systems together in some cases. We describe herein the design and methodologies of both forward and reverse genetic screens using different *PB* constructs we have developed (Fig. 1). While most of the considerations in the experimental designs make use of the traits unique to *PB*, these constructs have nested *SB* terminal repeats to yield maximal flexibility in the experimental design.

We have previously described the direct co-electroporation of a plasmid containing the *PB* transposase with a second plasmid containing the *PB* transposon as an efficient way of transducing mouse embryonic stem (ES) cells 2–3 orders of magnitude higher than *SB11*, with approximately 28% of cells that survive electroporation transduced [7]. Transposase is transiently expressed from the ‘helper’ plasmid, excising the transposon from the ‘donor’ plasmid and integrating it randomly into the genome. Depending on the experimental design, we also previously described the construction of *mPB-L3-ERT2*, which is a constitutively expressed transposase that is inactive until the addition of its chemical inducer 4-hydroxytamoxifen (4-OHT) [5]. This gives further flexibility in the control of transposition. In comparison, standard techniques for transgenics such as knock-in gene targeting are useful where a single copy of transposon and defined locus is required. Pro-nuclear injection will generate concatemers at random insertion sites and also result in transgenic lines with variable copy numbers, but lack the flexibility of being able to genotype and select for clones of interest before establishing the animal line.

*PB* transposition is unaffected by cargo sizes up to 9.1 kb, showing some reduction in efficiency at 14.3 kb [6]. Over-production inhibition (OPI) is a phenomenon that affects most transposable elements where increasing cellular transposase concentration beyond a certain threshold decreases transposition efficiency, though different studies have described both the presence and lack of OPI for *PB* [9,10]. Although we have not observed OPI in our experiments, when working with a new cell line, it is advisable to try several combinations of transposase and transposon concentrations to assay efficiency. Since all that is needed for transposase mediated integration of the transposon cargo into the genome is the introduction of the two DNA plasmids into the cell, the actual method of DNA delivery itself is quite flexible. We routinely use electroporation, transfection or nucleofection in both murine and human cells, the selection of the delivery system is dependent on the particular cell type in question.

Another consideration in utilising transposons is the presence of local hopping. During transposition, DNA transposons often have a propensity of reintegrating near the original excision site, a certain percentage of transposition events will be local and the rest would usually be distributed evenly across the genome, subject to other biases and tendencies. In mammalian cells, *PB* exhibits approximately either low (approximately 10%) or no reported local hopping (this is possibly context or loci dependent), and had a narrow local hopping distribution of approximately 100 kb from the donor site [6,7,11]. The rest of the insertion events seem random and evenly distributed across the genome. By comparison, *SB* is reported to exhibit local hopping in the range of approximately 10–75% contingent on the tissue and cellular context, with a distribution in the region of 3–6 mb from the donor site [12–15]. By using either or both systems, a library of desired distribution and saturation can be created. Chromosome engineering and screen strategies that harness the local hopping traits of transposon have been described in both insect and mammalian systems [16,17].

### 1.2 Designing *piggyBac* genetic screens

For mammalian models, point mutagens such as *N*-ethyl-*N*-nitrosourea (ENU) and insertional mutagenesis by viruses have been used with great success in diverse applications, though there were also inherent limitations. These range from the scale and cost of recovering mutations (for ENU) to viral tropism and insertion site biases within the genome.

The *Sleeping Beauty* transposon system has been used successfully for somatic insertional mutagenesis and cancer gene discovery in multiple tissues [13,18,19]. We have found that the *piggyBac* system is complementary to *Sleeping Beauty* in terms of the novel cancer genes identified [20]. The design of the transposon construct depends on the kind of genes to be identified from the screen. We have constructed three designs of transposons for insertional mutagenesis – bi-functional transposons that both inactivate local transcripts and ectopically activate surrounding genes, inactivating transposons that inactivate local transcripts, and activating transposons to drive expression of nearby genes (Fig. 1A–D). It is also possible to include a sensitising or cooperative transgene within the transposon itself (Fig. 1D), this makes use of the larger cargo capacity of the *PB* transposon and is not dependent on the availability of an existing mutant mouse strain. It allows both the delivery of sensitising mutations that are gain of function from expression of a cDNA as well as knockdown of a tumour suppressor gene using shRNAs. Multiple combinations of sensitising alleles can be assembled by using T2A junctions between cDNAs, so that two or more cDNAs are expressed from the promoter [21].

The considerations for choice of transposon design in a cell-based phenotypic screen are similar. The expression of *PB* transposase would usually be transient in such an experiment (see Section 3.1), so the saturation and genome coverage of the screen would be dependent on the number of cells bearing transposons. The transduction efficiency when starting work on a new cell line should be assayed using a selectable control transposon such as *PB-SB-PGK-Neo*.

Given the flexibility of transgene delivery for *PB*, it is easily adapted for the construction of a cDNA expression library for reverse genetic screens (Fig. 1E and F). We have cloned collections of cDNAs and shRNAs into the transposon vectors by PCR-cloning. For cell-based experiments, an overexpression construct (Fig. 1E) with a promoter such as CAGG (synthetic cytomegalovirus [CMV] early enhancer and chicken β-actin promoter hybrid) or PGK (phosphoglycerate kinase promoter) is used to deliver genes of interest into cells. In addition to assaying a single gene/construct per experiment, a library pool of transposons can be used in a single electroporation, resulting in a heterogeneous mixture of cells. Selecting for the phenotype of interest would then allow the recovery of the transposon or combination of transposons responsible for the cellular phenotype. We have also applied the pooled transposon approach to validating cancer mutations identified in human cancer genomes. We cloned a collection of mutant cDNAs into a promoter trap transposon (Fig. 1F) and generated animals that stochastically express the mutant cDNAs *in vivo*. Oncogenic mutant cDNAs transposons are recovered by analysing the transposons that are expressed in tumours that arise in these animals. Comprehensive cDNA and shRNA libraries constructed in the PB transposon will enable prospective genetic screens.

## 2 Materials

We have previously described both the *PB* transposase sources and the original vector backbone of the *PB*–*SB* hybrid transposon used to generate subsequent transposon constructs [5,7]. Published materials from us are available at http://www.sanger.ac.uk/technology/clonerequests/.

### 2.1 Transposase source

1. *CAGG-PBase* plasmid construct – PL622 is a plasmid source of *PB* transposase driven by the constitutive *CAGG* promoter (synthetic cytomegalovirus [CMV] early enhancer and chicken β-actin promoter hybrid).
2. *Rosa-PBase* knock-in mouse line – the knock-in *Rosa-PBase* allele was targeted and characterised in AB2.2 mouse embryonic stem (ES) cell line, and subsequently used to generate the PLPB mouse line by blastocyst injection.

### 2.2 Plasmid constructs

Fig. 1 outlines a catalogue of transposon constructs for different uses. We incorporate unique sequence tags in the 3′ end of the expression constructs to facilitate transgene tracking and differentiation of the introduced cDNAs from an endogenous expression. We have used both standard techniques and throughput scalable In-fusion Advantage PCR cloning kit (Clontech 639620) to generate constructs. Plasmid DNA is prepared using maxiprep plasmid purification kits (Qiagen 12163), resuspended at 1 μg/ml and stored at −20 °C.

1. Bi-functional activating/inactivating (Fig. 1A) – this transposon design causes loss of function to genes it lands in by either direct insertional disruption of the coding sequence or by trapping transcripts via the splice acceptors in both orientations. It also mediates ectopic gain of function by directional activation of nearby genes from the promoter and splice-donor.
2. Inactivating (Fig. 1B and C) – these transposons causes loss of function to genes it lands in by either direct insertional disruption of the coding sequence or by trapping transcripts via the splice acceptors in both orientations. The β-geo (β-galactosidase and neomycin phosphotransferase fusion) cassette provides a reporter readout for the expression pattern of the mutated genes.
3. Activating (Fig. 1D) – this transposon design activates nearby genes from its promoter and splice donor. Additional sensitising or cooperating transgenes can be added via an expression cassette within the construct.
4. Gene expression (Fig. 1E) – this is a gene expression construct using expression of transposase to mediate insertion into the genome.
5. Promoter/enhancer trap (Fig. 1F) – this transposon design traps transcripts from genes it lands in/near to, and expresses its payload cDNA through an internal ribosomal entry sequence (IRES). This assays the phenotype associated with a cDNA upon stochastic and ectopic expression *in vivo*.

### 2.3 Transposon delivery into cells

1. 10 cm diameter tissue culture dishes (Corning 430167).
2. 96-well round bottom tissue culture plate (Corning 3790).
3. 24-well tissue culture plate (Corning 3524).
4. M15 medium – Knockout DMEM (GIBCO 10829018) + 15% FBS (Invitrogen 10108165), 1× PSG (Penicillin, Streptomycin and Glutamine, GIBCO 10378016), 1× Non-Essential Amino Acids (GIBCO 11140035), 0.1 mM β-mercaptoethanol (Sigma M6250), 1000 U/ml murine or human LIF (Millipore ESG1107, LIF1010).
5. M10 medium – Knockout DMEM (GIBCO 10829018) + 10% FBS (Invitrogen 10108165), 1× PSG (Penicillin, Streptomycin and Glutamine, GIBCO 10378016), 1× Non-Essential Amino Acids (GIBCO 11140035).
6. Dulbecco’s phosphate buffered saline (PBS, Invitrogen 14190169).
7. 0.05% Trypsin–EDTA (GIBCO 25300054).
8. Centrifuge (Eppendorf 5810).
9. Gene Pulser electroporation cuvette, 40 mm gap (Bio-Rad 165-2088).
10. Gene Pulser Xcell electroporation system (Bio-Rad 165-2660).
11. Geneticin selective antibiotic liquid (Invitrogen 10131-027).
12. 0.1% (w/v) gelatin in sterile water, autoclaved (Sigma G9136).
13. Mitotically inactive SNL76/7 feeder cells (cell line and its associated protocols are available from Sanger Institute, see above. Also available at ATCC/LGC standards, http://www.lgcstandards-atcc.org/ or http://www.atcc.org).
14. Dimethyl Sulfoxide (DMSO, Sigma D2650).
15. MEF nucleofector kit 2 (Lonza VPD-1005).
16. Nucleofector Device (Lonza AAD-1001).

### 2.4 Mapping insertion sites by splinkerette PCR

1. DNeasy blood and tissue kit (Qiagen 69506).
2. Sau3A I restriction enzyme (New England Biolabs, NEB R0169L).
3. Thermal cycler (MJ Research PTC240).
4. Extensor Long PCR Master Mix, Buffer 1 (ABgene AB-0792/A).
5. QIAquick PCR Purification Kit (Qiagen 28106).
6. pGEM-T Easy Vector (Promega A1360).
7. Library Efficiency DH5 alpha Competent Cells (Invitrogen 18263012).
8. QIAprep Spin Miniprep Kit (Qiagen 27106).

## 3 Methodology

### 3.1 Transposon delivery into cells

For transposon delivery into mouse embryonic stem (ES) cells, we use the following electroporation protocol. This basic protocol is routinely adapted and optimised for other cell lines and delivery methods. In this example, the *piggyBac* transposon is used to efficiently deliver transgenes into the genome. Cells containing the transgenes are selected for using a Neomycin resistance cassette that is introduced either in the transgene constructs themselves or by co-electroporation of a *PB-SB-pGK-Neo* construct. After transgene delivery into mouse embryonic stem (ES) cells and genotyping for the desired clones, they can be made into mouse lines by blastocyst injections.

1. A 10 cm dish of ES cells at approximately 80% confluency is fed 2–4 h before being trypsinized for electroporation.
2. For each electroporation, prepare plasmid DNA mix as follows, 2 μg of PL622 *PB* transposase plasmid and 20 μg of transposon plasmid DNA.
3. Aspirate media from the 10 cm plate with ES cells, wash the dish with 10 ml PBS, aspirate PBS, repeat wash.
4. Trypsinise cells by adding 2 ml 0.05% trypsin/EDTA, swirl to cover entire surface. Place back in incubator for 15 min.
5. Add 2 ml M15 media and titurate to dissociate cells thoroughly.
6. Pellet cells by centrifuging for 5 min at 200 RCF in a centrifuge (1000 rpm) and resuspend the cells in PBS to a density of 1.1 × 10^7^ cells/ml.
7. For each electroporation, pipette 900 μl of cell suspension (10^7^ cells) into the tubes from (Step 2) containing DNA.
8. Pipette the DNA/cell mixture into a Gene Pulser electroporation cuvette, avoid touching the metal plates.
9. Electroporate the cells by placing the cuvette in the electroporation holder of the Bio-rad GenePulser. Set the machine to 230 V, 500 μF, time constant is expected to be between 5.6 and 8.0.
10. Transfer the cells gently from the cuvettes into 9 ml M15. Seed 1000 cells in a 10 cm dish with M15 media.
11. To select for cell clones containing the transgenes, replace with M15 media supplemented with 125 μg/ml Geneticin after 24 h. Colonies will be visible to the naked eye 7–10 days later. The media would need to be replaced daily.
12. To pick clones, aspirate media from the dish, wash the plate with 10 ml PBS, aspirate PBS, replace with 7 ml PBS.
13. Pick the colony up from the plate using a 20 μl pipette set to 4 μl. Dispense the cell clump in 50 μl of 0.05% trypsin/EDTA in a 96-well round bottomed plate. Incubate at 37 °C for 10 min.
14. Add 50 μl M15 to each well and titurate to dissociate thoroughly. Seed cell suspension to a fresh 24-well plate with M15 media.
15. Cell clones isolated can be used for further expansion, genotyping and subsequent injection into blastocysts to generate animal lines by standard techniques.

For transposon delivery into mouse embryonic fibroblasts (MEF) cells, we use the following nucleofection protocol with the MEF nucleofector kit 2. In this example, different combinations of expression constructs are tested for their ability to reprogramme MEFs into clonogenic induced pluripotent stem cells (iPSCs).

1. Preparation of SNL76/7 feeder cells (feeders should be seeded in the culture dishes 1–3 days before use).
  1.1 SNL76/7 cells are cultured in gelatinised tissue culture dishes with M10 media. Prepare gelatinised plates by coating 10 cm dishes with 0.1% gelatin solution for 30 min at room temperature. Aspirate before use.
  1.2 When cells are confluent, mitotically inactivate the cells by treatment with 10 μg/ml mitomycin c M10 media for 2 h.
  1.3 Wash twice with PBS, aspirating completely between washes. Cells can be used immediately or frozen down in large batches in M10 + 10% DMSO.
  1.4 Thaw a vial of mitotically inactive SNL76/7 cells in 10 ml M10 media.
  1.5 Pellet cells by centrifuging for centrifuge 5 min at 200 RCF (1000 rpm). Aspirate and resuspend gently in M10 media.
  1.6 Plate 4 × 10^6^ cells in each 10 cm dish. Allow cells to adhere at least overnight.
  1.7 Before use, aspirate M10 media and replace with M15 media.
2. MEF cells are grown in M10 media. Cells in a 10 cm plate are used when approximately 80% confluent.
3. For each electroporation, prepare plasmid DNA mix as follows, 1 μg of PL622 PB transposase plasmid and 2 μg of transposon plasmid DNA, and 100 μl of MEF II solution (from the nucleofector kit).
4. Aspirate media from plate, wash plate with 10 ml PBS, aspirate PBS.
5. Trypsinise cells by adding 4 ml 0.05% trypsin/EDTA, swirl to cover entire surface. Place back in incubator for 5–10 min until the cells lift off the plate.
6. Resuspend thoroughly by adding 4 ml M10 media and titurate to dissociate thoroughly.
7. Count cell numbers using a haemocytometer and for each nucleofection, centrifuge 10^6^ cells for 5 min at 200 RCF.
8. Resuspend cells in DNA + MEF II solution, and pipette into the kit cuvette.
9. Place cuvette into the Nucleofector device and use programme A-023, press ‘X’ on the Nucleofector device to start the programme.
10. Add 0.5 ml M15 media to the cuvette immediately and transfer cells gently into 10 cm culture dish using the sterile transfer pipette from the kit.
11. Colonies will be visible after 8–21 days. Colony numbers, cellular morphology and other assays for assessing reprogramming quality are scored.

### 3.2 *In vivo* genetic screen for cancer genes

**Fig. 2:**
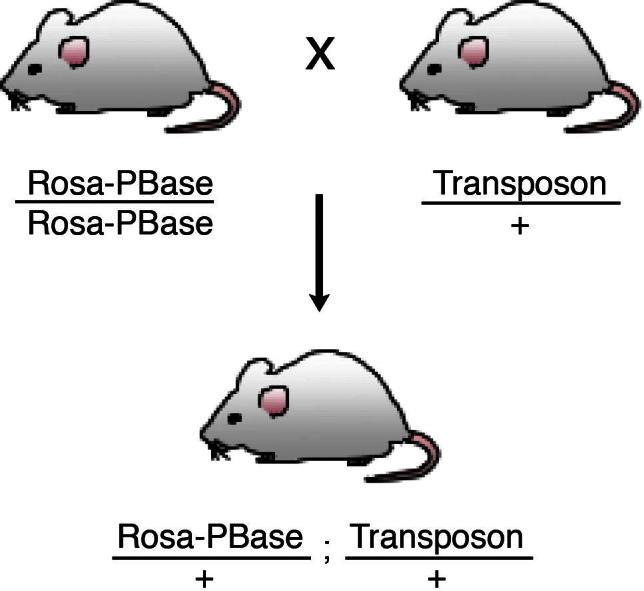
Typical genetic cross scheme for obtaining F1 experimental cohort mice carrying both the PB transposons and the transposase. The PLPB *Rosa-PBase* line used herein results in whole body somatic mobilisation of the transposons.

The general scheme for *in vivo* screening of cancer genes is to combine either activating/inactivating/bi-functional transposons with a source of transposase, the transposons will then translocate and stochastically activate or inactivate genes in somatic cells. Where these resultant mutation events are tumorigenic, the tumours that arise would have either oncogenes activated or tumour suppressor genes inactivated by the transposon, conferring a selective advantage to the clone. The cancer genes are identified by mapping the insertion sites of the transposons in the tumour genome to identify common insertion sites (CIS). Mouse lines containing transposons are crossed to a transposase-containing mouse line (Fig. 2). The progeny that contain both transposon and transposase undergoes somatic insertional mutagenesis, these comprise the experimental cohort for tumour watch. A screen to identify cancer genes using a bi-functional activating/inactivating transposon is described below.

**Fig. 3:**
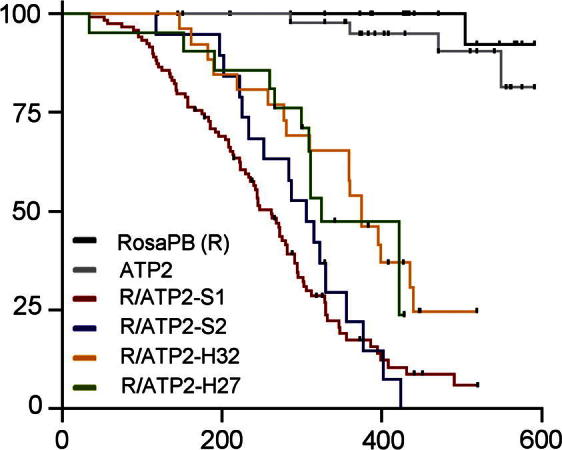
Survival curves of multiple ATP2 lines. Mice with ATP2 transposons and constitutive PB transposase expression show significantly decreased survival.

1. ATP2 construct – ATP2 transposon is generated in pBlueScript as bi-functional transposons that contain splice acceptors to trap surrounding transcripts and the MSCV (murine stem cell virus long terminal repeat) promoter to drive ectopic expression of nearby genes.
2. ATP2 mouse line – the ATP2 construct is used to generate founder lines by pro-nuclear injections. Successful founder animals are characterised for transposon copy numbers and the concatemer donor loci. Multiple ATP2 transgenic lines are established F2 mice, each with single transposon donor locus in the genome.
3. Generation of tumour watch animals – ATP2 lines are crossed to the constitutive *Rosa-PBase* line to generate the tumour watch cohort. The survival curves of these animals are shown in Fig. 3. Only animals with both transposon and transposase develop tumours with high penetrance, but not animals with only the transposon or transposase.
4. Identification of CIS and cancer genes – splinkerette PCR (see Section 3.4) is used to identify the insertion sites of transposons in tumours. The common insertion sites in the genome that occur more than which would be expected by chance are identified using previously described methods [22,23].

We have observed that both tumour spectrum and latency are related to the design of the transposon construct, specifically the kind of promoter used to drive ectopic expression of oncogenes [20]. ATP2 lines generated highly penetrant haemopoietic malignancies. In contrast, tumours driven by bi-functional transposon with the CAGG promoter were predominantly solid, and those with the PGK promoter were mixed.

### 3.3 *In vivo* validation of human cancer mutations

We previously described using a promoter trap version of an *SB* transposon that ectopically overexpresses promoterless cancer gene cDNAs *in vivo* [24]. With a constitutive transposase expression in the mouse line, the promoter trap transposons sample multiple developmental and tissue contexts in the whole body to assay the oncogenic potential of its cargo cDNA. Known human oncogenes produced murine tumours that recapitulated the spectrum observed in human patients. We have developed an updated design of the transposon (Fig. 1F) that requires less cloning steps and allows pools of candidate cancer mutations to be validated *in vivo*.

1. Enhancer trap constructs – candidate mutant cDNAs are identified from human cancer genomes. Both the wildtype and mutant cDNAs are generated and cloned into the enhancer trap transposon vector (Fig. 1F), a unique sequence tag 3′ of the stop codon in each cDNA facilitates ease of transgene genotyping and discriminating between endogenous vs transgene expression.
2. Generation of ES cell clones with pooled transposons transgenes – transposon constructs are electroporated into ES cells as a pool using the protocol in Section 3.1. The representation of each construct in a clone is detected by PCR using primers to the unique sequence tags. Activating transposition in animal lines with high transposon copy numbers can result in embryonic lethality. Using pools of 25 transposons per electroporation, we have recovered ES cell clones with up to 20 unique transposon insertions that give rise to chimaeras capable of germline transmission.
3. ES cell clones with multiple unique transposon integrations are injected into blastocysts to generate chimaeras using standard techniques.
4. Generation of tumour watch animals – chimaera animals are crossed to the constitutive *Rosa-PBase* line to generate the tumour watch cohort. As the transposons are introduced by pooled electroporation, the ES cell clones used to generate the chimaeras will have multiple insertion sites, the results in segregation of the transposons in the F1 tumour watch cohort. Each animal will have to be genotyped again to determine the compliment of transposons it has inherited. We have found highly variable latency and grades in the tumours that develop, depending on the oncogenic potential of the cDNA payload.
5. Recovery of tumorigenic mutant cDNAs – tumours that develop are assessed for the presence of transposon and for the expression of its cDNA payload. If an enhancer trapping transposon expresses an oncogenic cDNA in a suitable cell-type and developmental stage, it will drive the clonal expansion of the tumorigenic clone. Splinkerette PCR (see Section 3.4) is used to identify the insertion sites of transposons in tumours to establish clonal relationships between multiple tumours in an animal.

We have observed that in some bulk tumours, there is loss of multiple transposons compared to other normal tissues, and only a few highly expressed transposons remain. It is likely that transposons are lost through failure to re-integrate upon mobilisation in the dividing tumour cells, the retention of select transposons is indicative of purifying selection pressures to retain the driver oncogene cDNAs. We have not observed control non-oncogenic cDNAs expression at high levels in tumours, they are sometimes observed to be expressed at low levels in normal tissues due to stochastic enhancer trapping.

### 3.4 Mapping insertion sites by splinkerette PCR

To identify the insertion sites of the transposons in the genetic screens, we use a PCR-based splinkerette strategy that has been previously described [25]. A fragment of the transposon and adjacent genomic sequence is sequenced, allowing for mapping of the insertion site. The protocol as follows clones the insertion sites for sequencing. The number of colonies to be picked for sequencing depends on the transposon copy number in the genome, with more picked colonies allowing better recovery of the unique insertion sites.

Primers used in this protocol are

HMSpAa: CGAAGAGTAACCGTTGCTAGGAGAGACCGTGGCTGAATGAGACTGGTGTCGACACTAGTGG

HMSpBb: GATCCCACTAGTGTCGACACCAGTCTCTAATTTTTTTTTTCAAAAAAA

HMSp1: CGAAGAGTAACCGTTGCTAGGAGAGACC

PB-L-Sp1: CAGTGACACTTACCGCATTGACAAGCACGC

PB-R-Sp1: CCTCGATATACAGACCGATAAAACACATGC

HMSp2: GTGGCTGAATGAGACTGGTGTCGAC

PB-L-Sp2: GAGAGAGCAATATTTCAAGAATGCATGCGT

PB-R-Sp2: ACGCATGATTATCTTTAACGTACGTCACAA

1. Extract genomic DNA from tissues or expanded cell clones using DNeasy blood and tissue kit.
2. Digest 3 μg of genomic DNA with 4 U of Sau3A I in a reaction volume of 40 μl at 37 °C for 3 h, the enzyme is then deactivated at 65 °C for 20 min.
3. Generate the splinkerette adapter by annealing 150 pmol of HMSpAa and HMSpBb oligos together in 0.5× NEB buffer 2, final volume of 100 μl. The oligonucleotides are denatured at 65 °C for 5 min, then cooled to room temperature at −1 °C per 15 s using a thermal cycler. Annealed splinkerette stock is aliquoted and stored at −20 °C.
4. Ligate 750 ng (10 μl) of digested DNA to 4.5 pmols of splinkerette (3 μl) using 400 U T4 ligase (NEB M0202L) in final volume of 20 μl, incubate at 16 °C for 16 h, then inactivate at 65 °C for 10 min.
5. First round PCR is amplified with 5 μl of ligated DNA, 0.2 μM HMSp1, 0.2 μM PB-L-Sp1 (for mapping 5′ end of the transposon, use PB-R-Sp1 if mapping the 3′ end), 12.5 μl 2× PCR mix, final volume of 25 μl. PCR conditions are 94 °C for 1 min 30 s; then two cycles of 94 °C for 1 min, 68 °C for 1 min 30 s; then 30 cycles of 94 °C for 30 s, 65 °C for 30 s, 68 °C for 2 min; then 68 °C for 10 min.
6. Dilute the first round PCR product 1:100 with sterile water.
7. The nested PCR is amplified with 5 μl diluted 1st round PCR product, 0.2 μM HMSp2, 0.2 μM PB-L-Sp2 (for mapping 5′ end of the transposon, use PB-R-Sp2 if mapping the 3′ end), 12.5 μl 2× PCR master mix, final volume of 25 μl. PCR conditions are 94 °C for 1 min 30 s; then 30 cycles of 94 °C for 30, 60 °C for 30 s, 68 °C for 1 min 30 s; then 68 °C for 10 min.
8. Purify the nested PCR product using QIAquick purification kit. The purified product is ligated into the pGEM-T vector and transformed into DH5-alpha *E. coli* cells as per kit instructions. The resultant colonies are picked for minipreps and sequenced by standard primers such as T7 and SP6.
9. The sequencing results are analysed by aligning the flanking sequences to the genome to map the insertion sites of the transposons.
